# Early stage thymoma and the surgical extent paradigm

**DOI:** 10.1007/s13304-024-01918-z

**Published:** 2024-06-24

**Authors:** Gökhan Kocaman, Ayten Kayi Cangir

**Affiliations:** Thoracic Surgery Department, Faculty İbn-I Sina Hospital, Ankara University School of Medicine, 06100 Sıhhiye, Ankara, Turkey

**Keywords:** Thymoma, Thymic epithelial tumors, Complete thymectomy, Limited thymectomy

## Abstract

The recommended treatment for early stage thymoma without myasthenia gravis is complete thymectomy (CT). Limited thymectomy (LT) (simply resecting the thymoma with safe surgical margins) is gaining popularity. In this study, we compared the surgical and oncological results of complete and limited thymectomy in non-myasthenic patients with early stage thymoma. Non-myasthenic, Masaoka stage I–II, 86 patients who underwent surgical resection for thymoma were included in the study. Complete thymectomy (n:44) included patients who had resection of the thymoma together with the entire thymus and limited thymectomy (n:42) included patients who had resection of the thymoma without remaining thymus. The surgical approach, tumor size, histological type, pathological stage, adjuvant therapy, complications, postop myasthenia gravis, recurrence and death were recorded and compared between groups. Complete thymectomy group had more WHO type B1-3 tumors, more complications and more deaths than patients in the limited thymectomy group (*p* = 0.03, 0.018 and 0.023 respectively). Although statistically not significant CT group had more recurrences than LT group (11.4%/4.8%, *p* = 0.43). The 10-year freedom from recurrence (FFR) rate in the CT group was 84.8% and in the LT group ıt was 97.6%, the difference was not statistically significant (*p* = 0.15). None of the factors including surgical extent analysed with univariate and multivariate analysis had a significant effect on FFR. Limited thymectomy may be a good treatment option for non-myasthenic early stage thymoma patients but randomized controlled trials with long follow-up periods, ideally comparing patients operated with minimally invasive surgery are necessary.

## Background

Thymoma is the most common tumor of the anterior mediastinum in adults despite it’s low incidence. Surgery is the main treatment option in resectable cases [1]. The International Thymic Malignancy Interest Group’s (ITMIG) recommendation is complete thymectomy (CT) defined as resection of the thymoma together with the entire thymus for non-myasthenic thymoma patients and extended thymectomy for myasthenic patients [2]. There are several explanations why complete or even extended thymectomy is recommended in patients with non-myasthenic thymoma. These include the possible development of multicentric thymoma, occurrence of myasthenia gravis (MG) after thymectomy and reducing the possibility of local recurrence [3]. Multicentric thymoma is a rare condition and the role of extended thymectomy for controlling post-thymectomy MG is controversial [4]. It has been reported that similar oncological results can be achieved in non-myasthenic early stage thymomas by limited thymectomy (LT); simply resecting the thymoma with a safe surgical margin [5–12]. Limited thymectomy with minimally invasive methods are also reported to reduce postoperative complications, surgical trauma, and hospital stay [13].

In this study, we analysed the surgical and oncological results of patients operated for early stage thymoma without MG and compared CT and LT groups in terms of recurrence, complications and postoperative MG occurrence.

## Patient and methods

We retrospectively evaluated 261 patients operated for thymic epithelial tumors at our clinic from January 2000 to December 2022. Of these patients, 86 non-myasthenic, Masaoka Stage I-II thymoma patients who underwent curative surgical resection were included in the study. Patients with preoperative diagnosis of MG (n:93), Masaoka III-IV (n:62) tumors, thymic carcinomas or rare thymoma variants (n:8), debulking surgery (n:4), positive surgical margins (n:4) and patients who had neoadjuvant therapy (n:4) were excluded from the study.

All patients were evaluated preoperatively with thorax computed tomography. Most of the patients operated after year 2010 were evaluated with positron emission tomography and thoracic magnetic resonance imaging was used where needed. The histologic type of thymoma was classified according to the World Health Organisation (WHO) 2004 classification. Staging was performed according to the Masaoka staging. Surgical approaches included median sternotomy, thoracotomy and minimally invasive surgery (VATS: video assisted thoracoscopic surgery or RATS: robotic assisted thoracoscopic surgery). Postoperative complications were classified according to the Clavien-Dindo classification [14].

All the patients operated with median sternotomy had complete thymectomy (CT) and patients operated with other surgical approaches (thoracotomy, VATS, RATS) had limited thymectomy (LT). Operation types were choosen due to the clinical judgement of the principal surgeon. In the CT group (n = 44), the tumor was removed en-block with the entire thymus and in the LT group (n = 42) the tumor was removed with a safe surgical margin. Routine lymph node dissection wasn’t performed. The groups were compared according to age, sex distribution, surgical approach, tumor diameter, Masaoka stage, WHO classification, operation year, adjuvant treatment, complications and complication grade, postoperative MG, recurrence and death. Adjuvant therapy was mostly given to the Masaoka stage II tumors as radiotherapy. The site of recurrence was divided into three categories as local, regional and distant according to ITMIG’s recommendations. The patients were followed up with thorax computed tomography every 6 months for 5 years, and then annually for at least 10 years.

SPSS version 23.0 statistical software (IBM Corp., Armonk, NY, USA) was used for the data analysis. Data were expressed as mean, standard deviation (SD), median, interquartile range (IQR), percentage, minimum and maximum where appropriate. Groups were compared using the Chi-squared test and Fisher’s exact test for discrete variables. Mann–Whitney U test for non-parametric variables and Student’s *T* test for parametric continuous variables were used. The Kaplan–Meier method was used for survival analysis and survival differences were compared using the log-rank test. Freedom from recurrence (FFR) was calculated as the time period starting from the date of operation to the date of recurrence or last follow-up. Cox-regression analysis was used for univariable and multivariable survival analysis. A *p* value < 0.05 was considered statistically significant.

## Results

The study included 46 (53.5%) male and 40 (46.5%) female patients. The mean age of the patients was 52.3 ± 13.5 years (range 17–78 years) and 52 (60.5%) patients were symptomatic (chest pain, cough, etc.). Preoperative biopsy was performed in 12 (14%) patients. Median tumor diameter was 70 mm (IQR 25–75: 50–85.5 mm). Complete thymectomy with median sternotomy was performed for 44 (51.2%) patients and limited thymectomy with thoracotomy, VATS and RATS was performed for 42 (48.8%) patients. Invasive surgery was performed in 72 (83.7%) patients and minimally invasive surgery (VATS/RATS) in 14 (16.3%) patients. The median tumor diameter was 75 mm for patients who underwent invasive surgery (80 mm for patients operated via thoracotomy) and 29 mm for patients operated with minimally invasive surgery and the difference between them was statistically significant (*p* = 0.000). According to the WHO histological classification, AB was the most frequent type (n:39, 45.3%), followed by B1 (n:22, 25.6%), B2 (n:12, 14%), B3 (n:7, 8.1%) and A (n:6, 7%). Masaoka stages for the patients were stage I, 34 (39.5%); stage IIA, 47 (54.7%) and stage IIB, 5 (5.8%). Three (3.5%) patients developed MG in the postoperative period. Adjuvant chemotherapy was administered to 2 (2.3%) patients, radiotherapy to 36 (41.9%) patients and chemoradiotherapy to 7 (8.1%) patients. There was a significant difference in the ratio of patients who had adjuvant therapy operated before and after year 2010 (34.5%/61.4%, *p* = 0.018, respectively) and also more patients with Masaoka stage II tumors had adjuvant therapy than patients with stage I tumors (14.7%/76.9%, *p* < 0.001, respectively).

No complications were observed in 57 (66.3%) patients; 8 (9.3%) patients had phrenic nerve palsy, 2 (2.3%) patients had atrial fibrillation, 4 (4.7%) patients had wound infection, 1 (1.2%) patient had renal failure requiring hemodialysis and 4 (4.7%) fever, 1 (1.2%) chylothorax, 3 (3.5%) respiratory distress, 2 (2.3%) sternal separation, 1 (1.2%) Hb decrease requiring red blood cell transfusion, 1 (1.2%) venous thrombosis, 1 (1.2%) prolonged air leak and 1 (1.2%) pleural effusion were observed in patients. The most serious complications, one for each patient was graded according to the Clavien-Dindo (CD) classification and 9 (10.5%) patients had complications with grade I, 12 (14%) with grade II, 3 (3.5%) with grade IIIA, 3 (3.5%) with grade IIIB, and 2 (2.3) with grade IVA.

Median follow-up time was 75.3 months (min–max:9–190 months). Patients who had follow-up time over 10 years was 24.4% (n:21). Tumor recurrence was seen in 7 (8.1%) patients; 2 local, 2 regional and 3 distant recurrences. All the recurrences were seen among patients operated before year 2010 (*p* = 0.000). WHO type B1-3 tumors had three times higher recurrence rates than type A + AB tumors although statistically not significant (12.2%/4.4%, *p* = 0.25, respectively). There were 24 (27.9%) deaths and only 5 of them were thymoma related deaths.

Patients are grouped as complete thymectomy (CT) and limited thymectomy (LT) and the patient characteristics for these groups are summarized in Table 1. CT group had more WHO type B1–3 tumors, more complications and more deaths than LT group (*p* = 0.03, 0.018 and 0.023 respectively). Although statistically not significant CT group had more recurrences than LT group (11.4%/4.8%, *p* = 0.43). More patients had LT after year 2010 possibly due to increasing use of minimally invasive surgery (before and after year 2010 LT: 37.9%/% 54.4, *p* = 0.15, respectively).

**Table 1:** Patient characteristics

Characteristics N, (%)	Complete Thymectomy 44 (51.2)	Limited Thymectomy 42 (48.8)	*P*
Sex			0.5
Female	22 (50)	18 (42.9)	
Male	22 (50)	24 (57.1)	
Age, mean (± SD)	51.9 (13.4)	52.9 (13.8)	0.73
Surgical approach			**0.000**
Median sternotomy	44 (100)	–	
Thoracotomy	–	28 (66.6)	
Min. İnvz. Surg	–	14 (33.4)	
Operation year			0.15
≤ 2010	18 (40.9)	11 (26.2)	
> 2010	26 (59.1)	31 (73.8)	
Tumor diameter, median (min–max)	70 (30–230)	75 (15–180)	0.65
WHO type			**0.03**
A–AB	18 (40.9)	27 (64.3)	
B1–3	26 (59.1)	15 (35.7)	
Masaoka stage			0.53
I	16 (36.4)	18 (42.9)	
II	28 (63.6)	24 (57.1)	
Adjuvant therapy	20 (45.5)	24 (57.1)	0.27
Complications	20 (45.5)	9 (21.4)	**0.018**
Complication grades^a^			1
I–II	14 (70)	7 (77.8)	
IIIA–IVA	6 (30)	2 (22.2)	
Postop MG	1 (2.3)	2 (4.8)	0.61
Recurrence	5 (11.4)	2 (4.8)	0.43
Site of recurrence			
Local	1	1	
Regional	2	–	
Distant	2	1	
Death	17 (38.6)	7 (16.7)	**0.023**
Thymoma related deaths	5	–	
Follow-up time, median (min–max)	82 (9–182)	62 (12–190)	0.12

Bold values indicate statistically significant *p* values

*MG* myasthenia-graves, *Min. İnvz. Surg* minimally invasive surgery (VATS/RATS)

^a^Clavien-Dindo classification

**Fig. 1:**
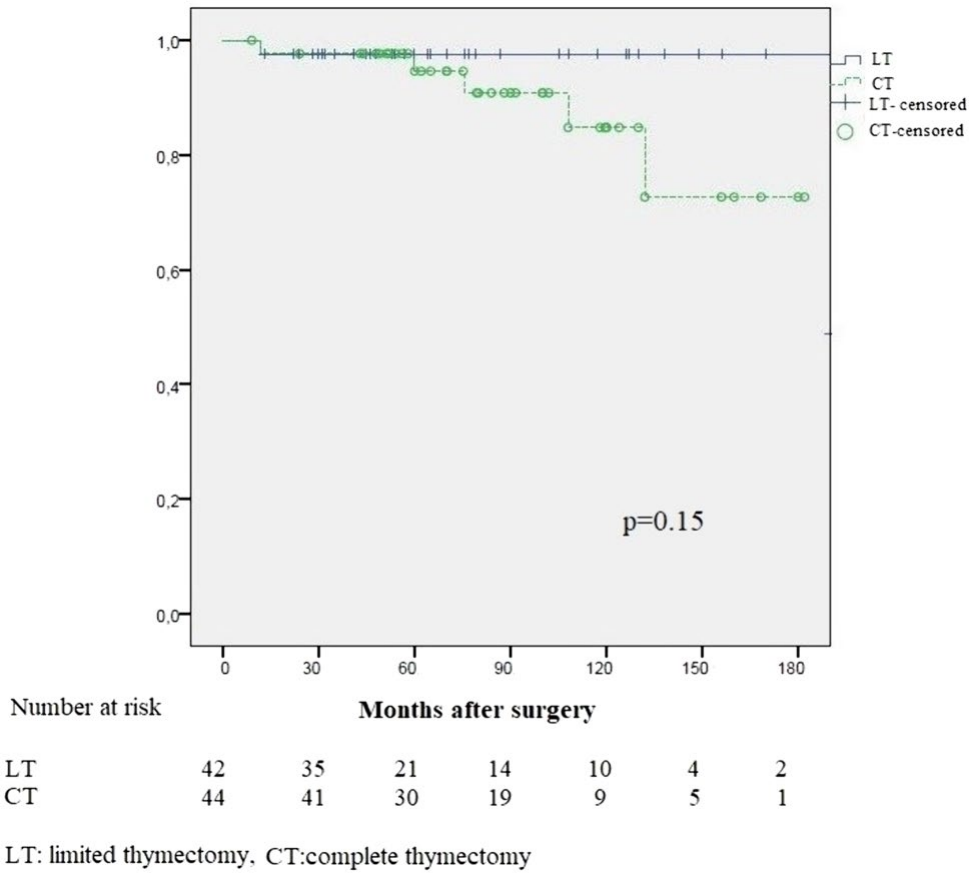
Freedom from recurrence curves for limited thymectomy and complete thymectomy

Five and 10-year freedom from recurrence (FFR) rates were 95.8% and 90.3% for the entire cohort. The 10-year FFR rate in the CT group was 84.8% and in the LT group ıt was 97.6%, the difference was not statistically significant (*p* = 0.15) (Fig. 1). The results of univariate and multivariate Cox regression analysis are summarized in Table 2. None of the factors analysed including surgical extent had a significant effect on FFR.

**Table 2:** Univariable and multivariable survival analysis

Characteristics	Univariable		Multivariable	
	HR (95% CI)	*P*	HR (95%CI)	*P*
Age	0.98 (0.93–1.04)	0.50	1.02 (0.92–1.12)	0.73
Female versus male	0.68 (0.15–3.07)	0.62	0.75 (0.05–10.98)	0.84
CT versus LT	0.23 (0.02–2.03)	0.19	1.04 (0.06–17.28)	0.97
Tumor diameter	0.98 (0.94–1.02)	0.44	0.98 (0.93–1.03)	0.52
WHO type A–AB versus B1–3	3.17 (0.59–16.95)	0.17	2.90 (0.19–43.74)	0.44
Masaoka I versus II	1.06 (0.23–4.81)	0.93	2.31 (0.12–44.30)	0.57
Adjuvant therapy	0.43 (0.08–2.24)	0.32	0.22 (0.01–4.77)	0.33

*CT* complete thymectomy, *LT* limited thymectomy, *WHO* World Health Organisation

## Discussion

There is still an ongoing debate about the surgical extent of early stage thymoma without MG and there are two main situations that cause this debate. Firstly, due to the increasing use of imaging methods, thymomas are detected in smaller sizes today and limited resections with minimally invasive methods are preferred for these small thymomas [7, 15]. In our study median tumor diameter of thymomas operated with minimally invasive surgery was 29 mm and all of them had limited thymectomy. The second and less discussed situation is limited resections performed via thoracotomy for large and lateralized thymomas. As seen in large series, most of the thymectomies performed with thoracotomy are limited thymectomy because it is technically difficult to perform total thymectomy with thoracotomy [5, 6, 12]. Also in our study median tumor diameter for patients operated with thoracotomy was 80 mm and all of them had limited thymectomy.

In the study of Bae et al. [15] published in 2014 including Masaoka stage I–IV thymomas; 63.4% of the patients without MG had limited thymectomy. In our study although including only Masaoka stage I and II thymomas, 48.8% of the patients had limited thymectomy. In the same study authors pointed out that although there was no difference between matched extended thymectomy and limited thymectomy groups in terms of freedom from recurrence rates, in the survival curves after 10 years there is an obvious decrease in the LT group. Also in our study, we didn’t find any significant difference between complete thymectomy and limited thymectomy groups in terms of freedom from recurrence rates (*p* = 0.15). All the recurrences were observed among patients operated before year 2010, only 24.4% of the patients had follow-up time over 10 years and more patients had LT after year 2010. We know that early stage thymoma recurrences occur much more later from the surgery. None of the studies in the literature don’t have median follow-up times over 10 years and limited thymectomy with minimally invasive surgery is a relatively new approach so for the most of studies there is a time bias favoring LT for recurrence including our study (Table 3).

**Table 3:** Literature summary comparing complete and limited thymectomy for recurrence rates

Author, year	Masaoka stage	Patient groups (n)	Median follow-up time (months)	Recurrence rate (%)	*P*
Sakamaki [9] 2008	I–II				NS
CT		19	64	0	
LT		11	26	0	
Odaka [10] 2010	I–II				NS
CT		18	58.6	0	
LT		22	21.6	0	
Onuki [11] 2010	I–II				
CT		61	67.3	0	
LT		18	104.2	5.5	**0.06**
Tseng [12] 2013	I–II				0.40
CT		42	62	4.5	
LT		53	55	1.9	
Bae [15] 2014	I–III	(matched)			NS
CT		86	94.5	5.8	
LT		86	85.6	11.6	
Nakagawa [5] 2016 (JART)	I–II	(matched)			0.10
CT		276	59	1.8	
LT		276	48	4	
Gu [6] 2016 (ChART)	I–II		NS		0.14
CT		796		3.1	
LT		251		5.4	
Narm [7] 2016 (KART)	I–II	(matched)			0.99
CT		141	50	3.5	
LT		141	48	4.9	
Tassi [4] 2017	I–IV				0.39
CT		70	84	2.9	
LT		22	43	0	
Voulaz [16] 2018	I–IV		77	14 (overall)	NS
CT		71	(overall)		
LT		86			
Rusidanmu [8] 2018	I–II		NS		0.45
CT		43		6.9	
LT		75		2.7	
Guerrera [17] 2021 (ESTS)	I (TNM)	(matched)	37 (overall)	NS	NS
CT		90			
LT		30			
Present study	I–II				0.43
CT		44	82	11.4	
LT		42	62	4.8	

Bold value indicates statistically significant *p* value

*ChART* Chinese Alliance for Research in Thymomas, *ESTS* European Society of Thoracic Surgeons, *JART* Japanese Association for Research on the Thymus, *KART* Korean Association for Research on the Thymus, *matched* propensity score matching used, *DFS* disease free survival, *NR* no recurrence, *NS* not stated, *TNM* eighth edition of the Union for International Cancer Control/American Joint Committee on Cancer tumour/node/metastasis stage classification, *CT* complete thymectomy, *LT* limited thymectomy

The recently published article by the European Society of Thoracic Surgeons (ESTS) Thymic Study Group claimed that thymothymectomy (CT) is superior to simple thymomectomy (LT) in terms of FFR [17]. However, in 12 other studies, including ours, no such superiority was demonstrated (Table 3). The median follow-up time of 37 months is too short to make this conclusion. The scarcity of LT patients in the ESTS group study is notable (32 patients, 6.4%) and the 5-year survival rate of 55% in the LT group for stage I thymoma patients is not consistent with the literature [18]. In the studies of the JART (Japanese Association for Research on the Thymus) [5], ChART (Chinese Alliance for Research in Thymomas) [6] and KART (Korean Association forResearch on the Thymus) [7] (with higher numbers of patients), the 5- and 10-year FFR rates are approximately 90% for both surgical groups. Also, in a meta-analysis, Papadimas et al. found no difference between patients who underwent limited or complete thymectomy in terms of recurrence, surgical margin positivity, adjuvant therapy and thymoma-related deaths. Complications, drainage time, and hospital stay were significantly lower in those who underwent limited thymectomy [13]. Interestingly, three studies including advanced stage thymomas, no statistically significant difference was observed between CT and LT groups in terms of recurrence [4, 15, 16].

Another issue about the limited thymectomy is surgical margin safety. There is no proven relation between the surgical extent and surgical margin positivity rate in the literature [4–7, 13, 16, 17]. Limited thymectomy doesn’t mean incomplete resection. In our cohort we had 4 R1 resections, 3 of them in the CT group and 1 in the LT group. We exclude these patients for FFR analysis.

Postoperative MG occurrence rate is about 1–4% [15]. There is not an evidence that shows complete thymectomy precludes postop MG development [13]. In our patient cohort 3 (3.4%) patients (1 patient in the CT group and 2 patients in the LT group) had postop MG. Another concern about the surgical extent is multifocal thymoma development. It’s rate is about 1%-3% and in our cohort there wasn’t any [15].

Postoperative complications seems to be higher for the complete thymectomy patients in the literature. Also in this study CT patients had more complications than LT patients but the severity of the complications were similar (*p* = 0.018, *p* = 1 respectively). Higher complication rates seems to be related with surgical approaches rather than surgical extent because most of the CT patients underwent sternotomy and LT patients were usually operated with minimally invasive techniques [4, 5, 7, 10, 12, 13, 16].

There are some limitations in our study. It is a retrospective, single center, long follow up study with a relatively low number of patients due to the nature of the thymomas because they are rare, slowly growing tumors and takes long times for recurrence. There may be selection bias between patient groups because the surgeon who performed the surgery decided on the type of surgical resection. Since the numbers of patients were similar in the two groups and the total number of patients was limited, propensity score matching could not be performed between the groups.

In conclusion, limited thymectomy may be a good treatment option for non-myasthenic early stage thymoma patients but randomized controlled trials with long follow-up periods, ideally comparing patients operated with minimally invasive surgery are necessary.

## Data Availability

The datasets used and/or analysed during the current study are available from the corresponding author on reasonable request.
